# Isoliquiritigenin suppresses human T Lymphocyte activation *via* covalently binding cysteine 46 of IκB kinase

**DOI:** 10.18632/oncotarget.11934

**Published:** 2016-09-10

**Authors:** Fenggen Yan, Fen Yang, Rui Wang, Xiao Jun Yao, Liping Bai, Xing Zeng, JiaJun Huang, Vincent Kam Wai Wong, Christopher Wai Kei Lam, Hua Zhou, Xiaohui Su, Juan Liu, Ting Li, Liang Liu

**Affiliations:** ^1^ State Key Laboratory of Quality Research in Chinese Medicine/Macau Institute for Applied Research in Medicine and Health, Macau University of Science and Technology, Macau, China; ^2^ Guangdong Provincial Academy of Chinese Medical Sciences, Guangzhou, China

**Keywords:** isoliquiritigenin, IKKβ, cysteine 46, T lymphocyte, immune-suppression

## Abstract

The efficacious practice of precision personalized medicine requires a more exact understanding of the molecular mechanisms of drug, hence then it is necessary to identify the binding site of the drugs derived from natural sources. In the study, we investigated the suppressive effect and underlying mechanism of isoliquiritigenin (2′,4′,4-trihydroxychalcone; ILG), a phyto-flavonoid, on human T lymphocyte activation *in vitro* and *in vivo*. The results showed that ILG dose-dependently suppressed human T cell activation *via* suppressing IκBα phosphorylation and degradation, NF-κB nuclear translocation and IKKβ activity. Molecular docking results predicted that cysteine 46 (Cys-46) is probably the binding site of ILG on IKKβ, and this prediction has been validated by competition assay and kinase assay. To further verify the binding site of this compound *in vivo*, IKKβC46A transgenic (IKKβ^C46A^) mice were generated. We found that ILG had a less potent immune-suppressive effect in homozygous IKKβ^C46A^ mice than IKKβ wild type (IKKβ wt) littermates with the delay-type hypersensitivity (DTH), suggesting that ILG cannot significantly suppress the inflammation due to the mutation of Cys-46 in the transgenic mice. Collectively, our findings indicate that the ILG inhibited T cell activation *in vivo* and *in vitro via* directly binding to IKKβ Cys46.

## INTRODUCTION

**Figure 1:**
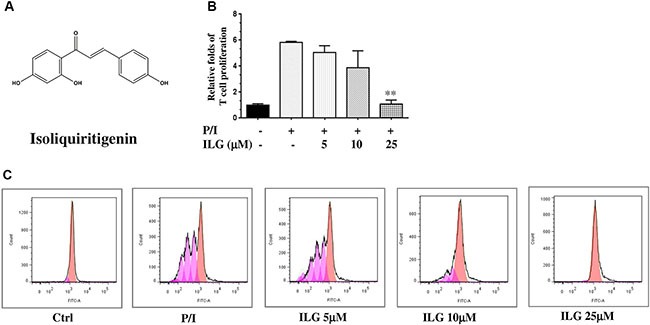
(**A**) The chemical structure of isoliquiritigenin (ILG). (**B**) The effect of ILG on human T cell proliferation induced by PMA plus ionomycin (P/I). (**C**) The effect of ILG on human T cell division induced by P/I. Statistically significant differences with respect to P/I are expressed as *P < 0.0*5* and **P < 0.01. Data represent the mean ± S.E.M. of three independent experiments.

With the advent of the Human Genome Project, the uniqueness of individuals and the importance of personalized medicine have been realized to provide more appropriate healthcare. However, it offers the challenges about identification of the accurate binding site of drugs. In the present study, the immunosuppressive effect, underlying mechanism and molecular binding site of isoliquiritigenin (ILG; 2′,4′,4-trihydroxychalcone (Figure 1A)) have been investigated on activated T lymphocytes *in vitro* and *in vivo*.

ILG, a member of the flavonoids, belongs to the chalcone family that is found in licorice, shallot and bean sprouts, and other medicinal herbs such as *Sinofranchetia chinensis*. The compound possesses various biological activities, including anti-inflammatory, antitumor and antihistamine effects *in vitro* and *in vivo* [1, 2]. It also induces apoptosis in human glioma cells, human hepatoma cells and gastric cancer cells [4–7]. In addition, ILG reduces H_2_ histamine receptor (H_2_R) activity and gastric acid secretion thereby protecting against gastric mucosal lesion formation in a pylorus-ligated rat model [8]. Regarding its anti-inflammatory activities, ILG markedly suppresses lipopolysaccharides (LPS)-induced prostaglandin E2 (PGE_2_) and cyclooxygenase-2 (COX-2) expression, nitric oxide (NO), interleukin-1β (IL-1β), and tumor necrosis factors-α (TNF-α) production, and induces heme oxygenase-1 (HO-1) expression through the extracellular signal-regulated kinase1/2 (ERK1/2) pathway in RAW 264.7 macrophages [9, 10]. Recently, it has been reported that ILG blocks TNF-α-induced expression of cell adhesion molecules in human endothelial cells by attenuating IκB kinase activity and ROS generation [11]. However, the effect of ILG on human T lymphocytes has not been well examined.

It is known that T lymphocytes play important roles in the development of autoimmune and inflammatory diseases including rheumatoid arthritis, contact dermatitis and systemic lupus erythematosus. In this connection, suppression of T cell activation is a dominant strategy in the clinical management of autoimmune disorders. T lymphocyte proliferation relies on NF-κB activation, which facilitates the production of a series of pro-inflammatory cytokines, thereby aggravating autoimmune disease activity [12]. IκB kinase β (IKKβ) is the key regulator of NF-κB, hence then IKKβ has become an attractive therapeutic target for developing new drugs for treating inflammatory and autoimmune diseases.

In the current study, we investigated the immunosuppressive effect of ILG *via* inhibiting NF-κB signaling in T lymphocytes. Molecular docking results predicted that IKKβ Cys-46 is probably the binding site of ILG. A competition assay and a kinase assay were performed to validate the virtual docking results. IKK^C46A^ transgenic mice were also generated and used to demonstrate that IKKβ Cys-46 is involved in the suppressive effect of ILG *in vivo*.

## RESULTS

### ILG inhibits human T lymphocyte proliferation and division

Because proliferation is one of the hallmarks of T cell activation, we first investigated the inhibitory effect of ILG on the proliferation of human T cells purified from human peripheral blood mononuclear cells (PBMC), The results clearly demonstrated that ILG dose-dependently blocked T cell proliferation generated by PMA plus ionomycin (P/I) from 5 to 25 μM (Figure 1B).

It has been reported that carboxyfluorescein diacetate succinimidyl ester (CFSE) could be used to monitor the number of cell divisions during proliferation and examine the relationship between proliferation and differentiation [13]. We therefore used CFSE to demonstrate the effect of ILG on cell division. The results showed that ILG significantly reduced the number of cell divisions at 10 μM and almost totally blocked the number of cell divisions at 25 μM (Figure 1C and Table 1).

**Table 1:** The effect of ILG on human T cell division induced by P/I

Measure of proliferation	Control	P/I	ILG (μM)		
			5	10	25
% Divided	0.033 ± 0.014	0.437 ± 0.065	0.319 ± 0.063	0.120 ± 0.019^*^	0.011 ± 0.004^*^
Div. Index	2.382 ± 1.489	29.770 ± 3.896	22.600 ± 3.470	9.343 ± 1.583^*^	0.393 ± 0.179^*^

Statistically significant differences with respect to P/I are expressed as *P < 0.0*5* and **P < 0.01. Data represent the mean ± S.E.M. of three independent experiments

### ILG blocks IL-2 and IFN-γ secretion, as well as cell cycle progression in human T lymphocytes

**Figure 2:**
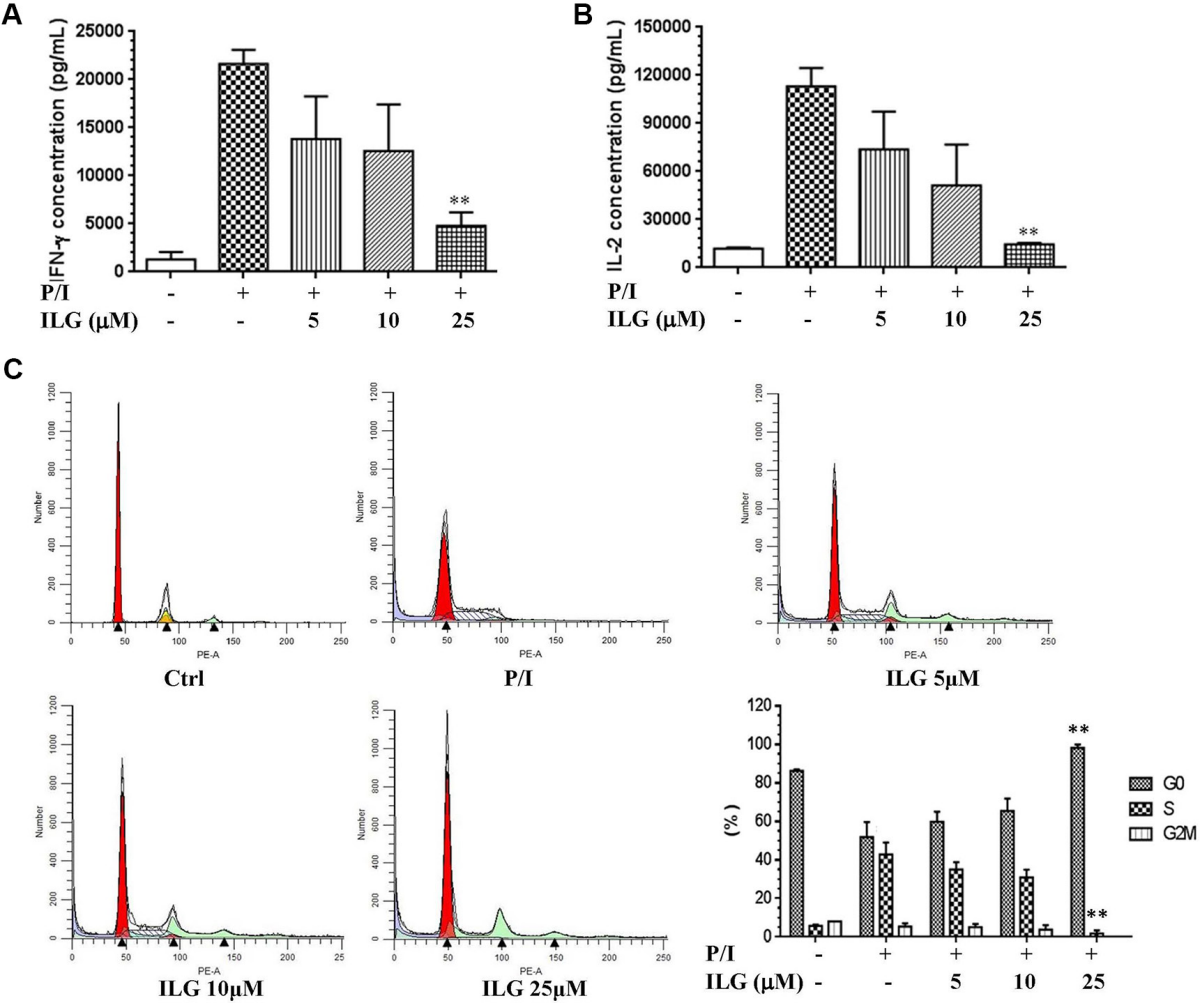
(**A**–**B**) The effect of ILG on IFN-γ and IL-2 secretion on human T cells induced by P/I. (**C**) The effect of ILG on the human T cell cycle progression. Statistically significant differences compared to vehicle treatment are expressed as *P < 0.05 and **P < 0.01. Data represent the mean ± S.E.M. of three independent experiments.

As mentioned above, T cell proliferation is one of the hallmarks of T cell activation; the other hallmark is the T cell growth factor secretion, including IFN-γ and IL-2. We therefore further investigated the effect of ILG on cytokine secretion. As shown in Figure 2A and 2B, ILG significantly and dose-dependently suppressed the expression of both cytokines, which were greatly induced by P/I in T lymphocytes. Cell cycle commitment determines cell proliferation and cytokine secretion, hence then we further examined the effect of ILG on the cell cycle progression. As shown in Figure 2C, cycling of P/I-mediated cells was progressing from G0/G1 to S and G2-M phase, whereas it was blocked at the G0/G1 phase by ILG at 25μM.

### ILG suppresses CD69 and CD25 expression on human T lymphocyte surfaces

**Figure 3:**
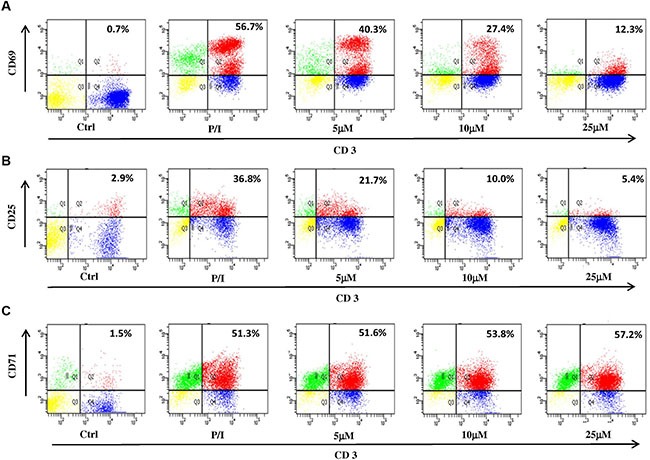
(**A**–**C**) The effect of ILG on the expression of CD69, CD25 and CD71 on human T lymphocytes induced by P/I. Values represent percentages of the double stained cells.

T cell surface activation markers, including CD69, CD25 and CD71, accompany the entry of T cells through G0/G1 to S phase. It has been demonstrated that ILG dose-dependently arrested cell cycle from 5 to 25 μM; therefore, we further investigated the effect of ILG on the expression of T cell surface activation markers. The results showed that the expression of CD69, CD25 and CD71 were 56.7%, 36.8% and 51.3% on human T cells stimulated with P/I, whereas ILG reduced the expression of CD69 and CD25 to 12.3% and 5.4% (Figure 3A–3B), respectively. Interestingly, we observed that ILG exhibited the opposite effect on CD71, showing it slightly up-regulated CD71 expression from 51.3% to 57.2% (Figure 3C).

### ILG inhibits IKK-IκBα-NF-κB signaling of human T lymphocytes

**Figure 4:**
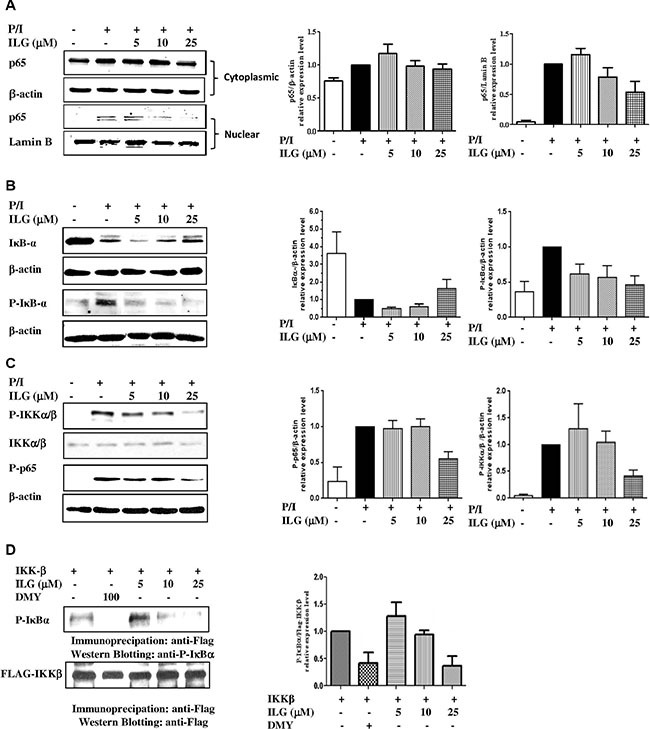
(**A**) The effect of ILG on the nuclear translocation of NF-κB subunit p65 in human T cells stimulated by P/I. (**B**) The effect of ILG on degradation and phosphorylation of IκBα in human T lymphocytes stimulated by P/I. (**C**) The effect of ILG on IKKα/β phosphorylation and p65 phosphorylation in human T lymphocytes stimulated by P/I. (**D**) The effect of ILG on IKKβ activity. Data are representative of three independent experiments.

NF-κB signaling plays a crucial role in T cell activation [14]. We therefore evaluated the effect of ILG on IκBα-NF-κB signaling on human T lymphocytes. Considering the pivotal role of the transcriptional factor in the pathway, we examined whether ILG could inhibit the expression of NF-κB p65 in the nucleus of T lymphocytes. As shown in Figure 4A, P/I stimulation could elevate p65 nuclear translocation, and ILG dose-dependently suppressed the p65 expression in the nucleus of T cells.

As p65 nuclear translocation is regulated by IκBα phosphorylation and degradation, we investigated whether the inhibition of NF-κB nuclear translocation results from a suppressive effect of ILG on IκBα phosphorylation and degradation. As shown in the upper panel of Figure 4B, ILG markedly suppressed IκBα degradation in a dose-dependent manner. To further determine whether the reduction of IκBα degradation was a result of the inhibitory effect ILG on IκBα phosphorylation, we used the proteasome inhibitor N-acetyl-leucyl-leucyl-norleucinal (ALLN) to block the degradation of IκBα, and treated the cells by P/I with or without ILG. The results showed that ILG strongly suppressed IκBα phosphorylation (Figure 4B, lower panel). In addition, it has been reported that stimulus-induced phosphorylation of IκBα regulated by the IKK could be rapidly degraded by an ubiquitin-26S proteasome [14]. Considering the inhibition effect of ILG on the phosphorylation and degradation of IκBα, we investigated the effect of ILG on IKK-α/β phosphorylation, and the results clearly showed that ILG significantly and dose-dependently suppresses IKKα/β phosphorylation (Figure 4C, upper panel). It was reported that IKKα regulates IKKβ kinase activity by phosphorylating IKKβ [15], and the effect of ILG on IKKα phosphorylation has been determined in the current study. Interestingly, ILG showed no significant effect on IKKα phosphorylation (Supplementary Figure S1), suggesting that the compound may have specific inhibitory effect on IKKβ phosphorylation.

Recent studies have demonstrated that the phosphorylation of p65 at Ser-536 by IKKβ contributes to NF-κB transcriptional activity [16]. Because ILG inhibits NF-κB nucleus translocation in an IκB-dependent manner, we investigated whether p65 phosphorylation suppressed by ILG is dispensable for NF-κB inhibition. The results show that 25 μM ILG almost completely abolished phospho-Ser^536^ p65 levels by using an antibody specific for phospho-Ser536 p65 (Figure 4C, lower panel). Hence then, we determined whether ILG directly influences IKKβ and then suppressed phosphorylation of p65 and IκBα by a kinase assay. As shown in Figure 4D, the substrate of IKKβ, IκBα, phosphorylation was suppressed by ILG as well as dihydromyricetin (DMY), the reference compound [18], implying that ILG inhibits IKK-IκBα-NF-κB signaling of human T lymphocytes *via* the regulation of IKKβ activity.

### ILG suppresses IKKβ activity *via* binding to Cys-46

**Figure 5:**
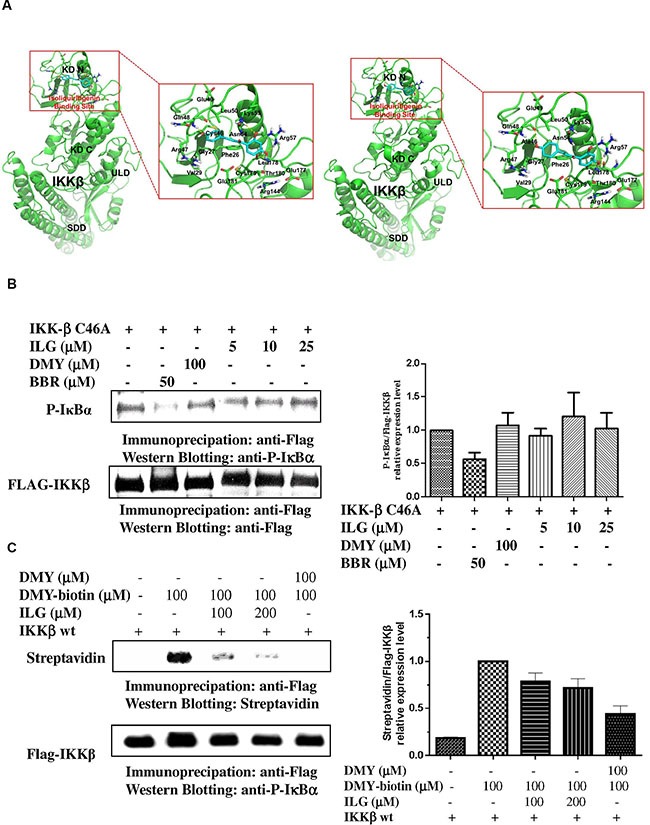
(**A**) Prediction of the molecular target of ILG by computational docking. High-precision computational model was applied to predict the approach that combines two elaborately built machine learning systems and multiple molecular docking tools to assess binding potentials of ILG against IKKβ involved in a complex molecular network. (**B**) The effect of ILG on the activity of IKKβ C46A. (**C**) Competitive binding experiments are used to elucidate the binding site of ILG at IKKβ. Data are representative of three independent experiments.

To determine whether ILG inhibits IKKβ activity *via* directly binding to IKKβ, we conducted molecular docking modeling to predict whether IKKβ is the molecular binding site of ILG. According to our results, the docking score of ILG with wild-type IKKβ and its C46A mutant are -12.23 and -11.76, respectively. Figure 5A illustrates the binding mode of ILG with IKKβ, showing that ILG probably binds to IKKβ by hydrophobic, polar, and hydrogen bond interactions at Cys-46.

In the wild-type system, the hydrophobic groups of ILG form hydrophobic interactions with the side chains of Phe26, Gly27, Val29, Cys46, Leu50, Leu178, Cys179, and Thr180. The polar groups of ILG exhibit polar interactions with the side chains of Arg47, Gln48, Glu49, Lys53, Asn54, Arg57, Arg144, Glu177, and Glu181 (Figure 5A, left panel). In addition, ILG forms hydrogen bonds with the backbone of Gln48 and the side chains of Lys53, Arg57, and Leu178.

In the C46A mutant system, the binding mode of ILG in IKKβ was changed due to the C46A mutation of the protein. The hydrophobic groups of ILG form hydrophobic interactions with the side chains of Phe26, Gly27, Val29, Ala46, Leu50, Leu178, Cys179, and Thr180. The polar groups of ILG exhibit polar interactions with the side chains of Arg47, Gln48, Glu49, Lys53, Asn54, Arg57, Arg144, Glu177, and Glu181. In addition, ILG forms hydrogen bonds with the backbone of Gln48 and Glu177 and the side chains of Arg57, and Leu178 (Figure 5A, right panel). Collectively, the molecular docking results suggest that IKKβ Cys-46 is probably the binding site of ILG.

To further verify whether the inhibitory effect of ILG on IKKβ activity is associated with targeting of Cys-46 of the protein, single point mutant IKKβ constructs in which Cys-46 is replaced with alanine (A) (IKKβ C46A) by site-directed mutagenesis were generated. The *in vitro* IKKβ kinase assay showed that IKKβ C46A has no response to ILG and DMY (Figure 5B). By contrast, berberine, which is reported to target IKKβ on Cys-179 [18], could suppress the IKKβ C46A activity, implying that Cys-46 is the target of ILG and DMY and not the target of berberine.

In our previous study, it was found that biotinylated-DMY (biotin-DMY) directly binds to IKKβ Cys46 *via* a covalent bond [18]. Considering the virtual computational docking results of ILG on IKKβ, we utilized DMY-biotin, the validated probe, to determine whether ILG binding IKKβ C46A relies on covalent bonds. Therefore, a competition assay was performed with reducing SDS-PAGE. As shown in Figure 5C the signal of DMY-biotin could be easily found, whereas the DMY-biotin signal was significantly reduced in addition of ILG. Increases in ILG amount resulted in less DMY-biotin signal, suggesting that the binding sites of ILG and DMY-biotin overlap. Collectively, these results show that IKKβ Cys46 is the molecular target of ILG *via* covalent binding *in vitro*.

### The anti-inflammatory effect of ILG was abolished in IKKβ^C46A^ transgenic mice

**Figure 6:**
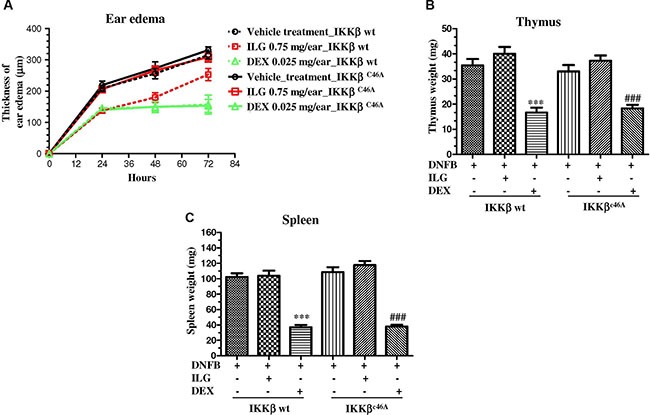
(**A**) The effect of ILG on the ear edema of the IKKβ^C46A^ transgenic mice and wild-type littermates with delayed type hypersensitivity (DTH). (**B**–**C**) The effect of ILG on thymus and spleen weight of DTH IKKβ^C46A^ transgenic mice and wild-type littermates. Statistically significant differences with respect to the vehicle treatment in IKKβ wt and IKKβ^C46A^ transgenic mice are expressed as *P < 0.05, **P < 0.01 and ***P < 0.001; ^#^*P* < 0.05, ^##^*P* < 0.01 and ^###^*P* < 0.001, respectively.

The above results demonstrated that ILG possessed the immune-suppressive effect on human T cells *via* covalent binding IKKβ Cys46 *in vitro*. To further elucidate whether the immune-suppressive effect of ILG on the T cell activation is the result of mediating Cys-46 of IKKβ *in vivo*, we generated IKKβ^C46A^ transgenic mice and conducted a delay-type hypersensitivity (DTH) experiment, a mouse inflammatory model for atopic dermatitis triggered by T cells. The results showed that the immune-suppressive effect of ILG was less potent in homozygous IKKβ^C46A^ mutant mice than in IKKβ wt mice (Figure 6A, Supplementary Table S1), which corroborates the *in vitro* results. The reference compound, dexamethasone, could significantly suppress edema and simultaneously induced the atrophy of thymus and spleen. Interestingly, we observed that ILG could not reduce thymus spleen weight in both IKKβ wt and IKKβ^C46A^ mutant mice (Figure 6B–6C). Collectively, these results suggest that IKKβ Cys-46 is the molecular target of ILG to suppress T cell activation *in vivo* without significant toxicity.

## DISCUSSION

Genomic variation plays an important role in the understanding of disease predisposition, biology and clinical response to therapy through effects on gene structure and expression. Advances in human genome research are opening the door to a new paradigm for practicing medicine that promises to transform healthcare [19]. Based on this concept, the personalized precision medicine, tailoring the practice of medicine to the individual, was recently initiated [19]. Under this premise, it is becoming more important to elucidate and verify the molecular target of the compounds being used for treatment. In recent years, medicinal plants are becoming an attractive source for drug discovery; for example, veregen, a mixture of flavonoids derived from medicinal plants, was approved by the FDA in 2006 to treat external genital and perianal warts. Although herbal medicines provide an extensive resource for the development of new drugs, the underlying mechanisms and molecular targets of the most compounds have not been well expounded. For example, pseudolaric acid B, which is derived from Chinese medicinal herb, has not been identified for its molecular target on the suppression on T cells [20]. ILG is a major chemical component of licorice (*Glycyrrhiza uralensis*), which is one of the most commonly used to “harmonize” other ingredients in Chinese medical formulas [21, 22], and ILG is effective in the prevention and treatment of inflammatory conditions [23–24]. However, the function of ILG in T cells has not been studied. Our current study is the first to report the immune-suppressive effect, underlying mechanism, as well as molecular target of the compound in T cells.

In the present study, we have shown for the first time that ILG, not only inhibits human T lymphocyte proliferation and division stimulated by PMA/ionomycin in a dose-dependent manner, but it also reduces the PMA/ionomycin-mediated human T lymphocyte cytokine secretion including IL-2 and IFN-γ, indicating that ILG could suppress T cell activation. We further found that ILG treatment could prevent cells from entering cell cycle, implying that ILG-induced cell cycle arrest might further contribute to the inhibition of T-cell proliferation and the production of the growth factors of T cells including IL-2 and IFN-γ. In addition, ILG could significantly attenuate CD69 and CD25 (IL-2 receptor) expression, and both of two markers were applied to evaluate the degree of immune responses. Interestingly, ILG slightly activates CD71, which is a marker of late stage T cell activation, implying that ILG probably has an influence on the early stages of T cell activation.

Because NF-κB suppresses CD25 expression, IL-2 production and T-cell proliferation, we further proposed that ILG might inhibit T-cell activation by blocking NF-κB signaling. Our expectation was confirmed that ILG could suppress the phosphorylation and degradation of IκBα, as well as NF-κB activity and phosphorylation of p65.

Since persistent activation of the NF-κB signaling pathway is often associated with many inflammatory and autoimmune diseases, we therefore conducted intensive investigations on this signaling with ILG intervention. IKKβ is a component of the IKK complex that serves as a protein subunit of IκB kinase, and plays a central role in the regulation of NF-κB signaling in response to a diverse set of extracellular stimuli [26]. According to literature reports, IKKβ harbors four binding sites, including ATP, Ser-177/181, Cys-179 and Cys-662/716 binding sites [27–30]. In our previous study, we found IKKβ can also offer a fifth binding site, Cys-46 of the protein [19], and it has been reported that a synthesized compound, ainsliadimer A, covalently binds to the conserved cysteine [31]. Ainsliadimer A could obviously inhibit IκBα phosphorylation and degradation at 8 μM in macrophage and cancer cell lines. Our current study demonstrated that ILG was able to significantly suppress NF-κB signaling in T lymphocytes at 25 μM. Although the different cells were employed in the studies, it could be speculated that ainsliadimer A has more potential than ILG to inhibit the NF-κB signaling.

Molecular docking programs are widely used as modeling tools for predicting ligand binding modes, as well as for a structure-based virtual screening approach [32]. To explore the binding site of ILG, we used computational tools for target identification and predicted that ILG probably binds to the Cys-46 of IKKβ. Furthermore, we conducted a kinase assay to demonstrate that mutation of IKKβ Cys-46 could abrogate the suppressive effect of ILG on the activity of IKKβ, which provided the evidence showing that Cys-46 of IKKβ is the binding site of ILG. Biotin-DMY has been identified binding to IKKβ Cys-46 in our previous study [18], and used as a probe to conduct a competition assay to demonstrate the molecular target of ILG on IKKβ. We found that berberine, targeting on the Cys-179 of IKKβ [17], still has the ability to inhibit the kinase activity of IKKβ C46A and wt, and the competition assay provided the evidence to show that Cys-46 is not the binding site of berberine on IKKβ. In concert with *in vitro* results, ILG has no significant immune-suppressive effect on IKKβ^C46A^ transgenic mice, whereas it shows significant inhibitory effect on IKKβ wt mice in a DTH animal model, which is mediated by T cells. Collectively, ILG alleviated IKKβ activity *via* binding on Cys-46 and then reduced the degradation and phosphorylation of IκBα, prevented NF-κB nuclear translocation, arrested human T cell cycle progression, cytokine secretion and T cell proliferation, eventually mediated the immune-suppressive effect on human T cells. In concert with *in vitro* results, the inhibitory effect of ILG was almost abolished completely in homozygous IKKβ^C46A^ mutant mice. In summary, all of our results clearly demonstrate that the immune-suppressive effect of ILG in T cells *in vivo* and *in vitro* results from binding on the IKKβ Cys-46.

Because the MAPK family plays a crucial role in triggering the immune response, we also examined the effect of ILG on the signaling. Our results clearly showed that ILG has no obvious effect on MAPKs signaling (Supplementary Figure S2), indicating that ILG has more intensive effects on IKKβ-NF-κB signaling than MAPKs. As ILG possesses a selective property on suppressing IKKβ activity, it is valuable to be further investigated on ILG as a lead compound into an immunosuppressive agent with clear molecular mechanisms for applications in inflammatory and autoimmune diseases in the future.

## MATERIALS AND METHODS

### Drugs and reagents

Isoliquiritigenin > 98% purity verified by HPLC was obtained from NanJing Zelang pharmaceutical R&D Co., Ltd. (NanJing, China). Pan T Cell Isolation Kit II was purchased from MACs (CA, USA). Anti-human fluorescein isothiocyanate (FITC)-CD25, FITC-CD69 and FITC-CD71 antibodies, as well as phycoerythrin (PE)-CD3 antibody and other antibodies including that against NF-κB, were purchased from BD Pharmingen Inc. (San Diego, CA, USA). Phorbol 12-myristate 13-acetate (PMA), flag immunoprecipitation kit and flag antibody were obtained from Sigma-Aldrich (St Louis, MO, USA). Ionomycin were obtained from Calbiochem( La Jolla, CA, USA). Cell proliferation kit was obtained from Roche (Roche, Basel, Switzerland). The primary antibodies used in the current study were rabbit antibodies specific for P-IκBα (Ser32/36), P-IKKα/β, P-JNK (Thr183/Try185), JNK, P-ERK1/2 (Thr220/Try204), ERK, P-p38 (Thr180/Try182), p38, IKK-α/β, p-IKKα/β, P-p65 (Ser536) and p-IκBα(Ser32) provided by Cell Signaling Technologies (Beverly, MA, USA); while mouse antibodies specific for IκBα (Cell Signaling, USA) and β-actin was provided by Santa Cruz (San Diego, CA, USA). Both IL-2 and IFN-γ ELISA kit, as well as carboxyfluorescein diacetate succinimidyl ester (CFSE) were bought from Life Technologies. All other common chemicals and reagents were from Abcam (Cambridge, MA, USA) or Sigma-Aldrich unless otherwise specified.

### Human T lymphocyte isolation, purification and stimulation

Human peripheral blood T lymphocytes were isolated from buffy coat according to the previous method [33]. In brief, the buffy coat provided by Macao Blood Transfusion Centre was mixed with normal saline, and then added to 50 ml centrifuge tube containing Ficoll-Pague plus (Amersham Biosciences (Piscataway, USA). The mixture was separated to several layers after centrifuged at 350 g for 35 min. The layer of mononuclear cells was collected, and purified by magnetic-activated cell sorting (MACs) pan T cell kit. 20 ng/ml PMA plus 1 μM ionomycin (P/I) was employed in each experiment with different time intervals for different purpose.

### T lymphocyte proliferation and cytotoxicity assay

The cell proliferation kit (Roche) was used to investigate the effect of ILG on the T cell proliferation according to the manufacturer's instruction. In brief, human T lymphocytes (1 × 10^5^/well) were cultured in 96-well plates in triplicate in RPMI 1640 medium plus 10% FBS, and then stimulated with 20 ng/ml PMA plus 1 μM ionomycin in the presence or absence of the compounds for 72 h. Before the cells were collected, BrdU was added to the cells at final concentration of 10 μM, and incubated for another 14 h. Finally, BrdU was determined by ELISA method according to the manual, and data were obtained from three independent experiments.

### Cell lines

The cell lines HEK293 (human embryonic kidney) was obtained from American Type Culture Collection. The HEK293 cells were cultured in DMEM supplemented plus 10% FBS. The culture medium was supplemented with 100 U/mL penicillin and 100 μg/mL streptomycin.

### Enzyme-linked immunosorbent assay

Enzyme-linked immunosorbent assay (ELISA) kit (Invitrogen, Carlsbad, CA, USA) was used to determine the amount of IL-2 and IFN-γ secreted by the activated human T lymphocytes. Briefly, cells (1 × 10^5^/well) were incubated in the presence or absence of ILG at different concentrations, For stimulated with PMA plus inomycin, the cells were pretreated with ILG for 2 h at different concentrations, and then stimulated with 20 ng/ml PMA plus 1 μM ionomycin for another 48 h. Finally, the culture supernatants were collected, and then concentration of IL-2 and IFN-γ in the supernatants was evaluated by ELISA method according to the manufacturer's instructions. Data were obtained from three independent experiments.

### T lymphocyte surface marker, intercellular protein, CFSE and cell cycle analysis

The expressions of T lymphocyte activation markers, including CD25, CD69 and CD71, were measured by flow cytometry according to the previously described method [34]. The cells (1 × 10^6^/well) was pretreated with ILG for 2 h, followed by stimulated with PMA (20 ng/ml) plus ionomycin (1 μM) [34]. To determine the expression of CD69, the cells were stimulated with PMA plus ionomycin for 24 h; to evaluate the expressions of CD25 and CD71, the cells were incubated with stimulators for 48 h. After collection, the cells were stained with indicated antibodies, and incubated for 30 min at room temperature avoiding from light, and then fixed with 4% paraformaldehyde (PFA). On the next day, samples were analyzed on FACS Calibur Flow Cytometer using CellQuest software. The separate tubes of cells stained with single-color antibodies for each of the flourochromes were severed as the compensation standards.

For analysis of cell cycle, human T lymphocytes (10^6^/well) were pretreated with ILG for 2 h followed by stimulated with or without PMA (20 ng/ml) plus ionomycin (1 μM) for 72 h. After collected, washed by PBS and fixed by 70% ethanol, the cells were stained by PI (Propidium Iodide, BD Pharmingen, San Diego, USA) for 30 min at room temperature, and then the cell cycle was analyzed by flow cytometry [35].

To monitor the number of cell divisions during proliferation, the division tracking dye carboxyfluorescein diacetate succinimidyl ester (CFSE) was used according to the previous method [36]. In brief, the cells were incubated with pre-warmed PBS/0.1% BSA at a final concentration of 1 × 10^6^ cells/mL. Two μL of 5 μM stock CFSE solution was added to the cells at final working concentration of 10 μM. After incubated with the dye at 37°C for 10 min, the cells were added ice-cold culture medium and incubated for 5 min to quench the staining. The cells were washed by fresh medium and treated with ILG for 2 h, followed by stimulation with PMA/ionomycin for another 5 days, and finally analyzed by flow cytometer.

### Analyses of cellular protein expressions by using Western blotting

To evaluate the phosphorylation form of IκBα, human T lymphocytes (4 × 10^6^/well) were pretreated with ILG at different concentrations followed by 100 μg/ml N-acetyl-leucyl-leucyl-norleucinal (ALLN) (Calbiochem, USA) for 60 min, and the cells were then incubated with PMA (20 ng/ml) plus ionomycin (1 μM) for another 60 min. For determination of IκBα, P-IKKα/b, P-p65 and b-actin from whole cellular proteins, the human T lymphocytes (4 × 10^6^/well) were stimulated with PMA plus ionomycin for 1 h after pre-incubated with different concentrations of ILG for 120 min. The T lymphocytes were harvested and lysed with lysis buffer (Sigma) with 1 × protease inhibitor mix (Roche) to prepare the whole cellular lysates. For NF-κB nuclear translocation assay, the cytoplasmic and nuclear fractions of T cells were prepared by using NE-PER™ Nuclear and Cytoplasmic Extraction (thermo fisher scientific, USA). The whole cellular or nucleus extracts were then subjected to electrophoresis in 10% SDS/PAGE and to immunoblotting according to the previous method [37].

### Transfection and immunoprecipitation

The transfection assay was preformed according to the manufacturer's instruction of lipofectamine LTX (invitrogen, USA). In brief, HEK293 cells were seeded in 1.5 ml of DMEM growth media plus 10% FBS at 5 × 10^5^ cells per well. Five hundred microliter Opti-MEM Reduced Serum Media containing 1.25 μg of DNA was added to the cells to be transfected, and then 1.25 μl of PLUS was added into the above diluted Opti-MEM:DNA solution, gently mixed and incubated for another 5 min at room temperature. Subsequently, lipofectamine LTX™ Reagent was added into the above solution, and then mixed gently and incubated 30 min at room temperature to form DNA-lipofectamine LTX Reagent complexes. Finally, 500 μl of the DNA-lipofectamine LTX Reagent complexes was directly added to each well containing cells and mixed gently. The cells were incubated at 37°C in a CO_2_ incubator for 24 h.

Immunoprecipitation was used to pull down IKKβ recombinant protein from HEK 293 cells overexpressing Flag-IKKβ according to the manufacture's instruction of Flag tagged protein immunoprecipitation Kit (Sigma). Briefly, HEK293 cells were harvested and lysed by incubation with lysis buffer for 15 min on ice, after transfected with Flag-IKKβ for 24 h. The lysate was collected by centrifugation for 10 min at 12,000 × g, cell lysates were then added to the resin provided by the kit. The resin was collected by centrifuging for 30 s at 8200 × g after agitated for overnight at 4°C. The Flag-IKKβ was eluted by competition with 3 × Flag peptide, and stored at −80°C or further conduct IKKβ kinase assay and competition assay.

### IKKβ kinase assay

To determine the effect of ILG on IKKβ activity, the IKKβ kinase assay was performed. Briefly, IκBα substrate supplied by Enzo Life Science (Farmingdale, NY, USA), Flag-IKKβ recombinant protein, and ATP were incubated with or without ILG at 30°C for 30 min. The mixture was analyzed by 10% SDS-polyacrylamide gel electrophoresis (SDS-PAGE), and then electro-transferred onto nitrocellulose membranes. The nitrocellulose membranes were incubated with P-IκBα (Ser32/36) for overnight at 4°C after blocked by 5% dried milk for 60 min. Next day, the membranes were further incubated with HRP-conjugated secondary antibodies for 60 min, and developed using ECL Western Blotting Detection Reagents (Life Technologies).

### Competition assay

Flag-IKKβ wild type (wt) was precipitated from HEK 293 overexpressing Flag-IKKβ, and incubated with ILG or DMY for 1h and then 100 μM DMY-biotin was added to the mixture. Subsequently, the proteins were separated by SDS-PAGE and transferred to nitro-cellulose membranes. After blocking with BSA and washing with PBST, the membranes were incubated with streptavidin horseradish peroxidase for 1 h and developed with enhanced chemiluminescence. Finally, the membranes were incubated with anti-Flag antibody to evaluate the expression of Flag-IKKβ.

### Computational methods

The initial 3D structure of ILG was built using the Molecule Builder module incorporated in MOE software. The structure was then subjected to energy minimization and partial charges calculation with Amber99 force field. The crystal structure of wild-type inhibitor of κB kinase β (IKKβ) was retrieved from Protein Data Bank (PDB ID code 3RZF [38]). On the basis of the wild type protein structure, the structure of IKKβ with C46A mutant was obtained by performing single point mutation with Rotamer Explorer in MOE software. To prepare the protein for molecular docking, the protein structure was subjected to partial charges calculation and energy minimization with Amber99 force field. Energy minimization was terminated when the root mean square gradient falls below 0.05 kcal/(mol·Å).

The prepared proteins and ligand were introduced for molecular docking. The docking site was identified by using Site Finder in MOE software. The identified binding site including residue Cys46 was chosen as the binding site for molecular docking according to our experiment. In molecular docking, the Triangle Matcher placement method and London dG scoring function were used. A total of 30 docking poses were generated for the ligand and the pose with the best binding mode was selected for further analysis.

### IKK-β^C46A^ transgenic mice

The IKK-β^C46A^ transgenic mice were generated by Shanghai Biomodel Organism Science & Technology Development Co., Ltd. (Shanghai, China). The transgenic mice were validated by PCR and gene sequencing. The IKK-β^C46A^ mice have been backcrossed to C57BL/6 for 6 generations in our experiments, and the wild-type littermates were served as control. They were kept under 12:12 h cycle of light with *ad libitum* access to food and drink. All mice were kept under specific pathogen-free conditions in the animal care facility at Guangdong Provincial Hospital of Traditional Chinese Medicine. Animal care and experiments were conducted in accordance with the Laboratory Animal Research Committee Guidelines of Guangdong Provincial Hospital of Traditional Chinese medicine.

### DTH animal model

Mice were sensitized through topical application onto their shaved abdomens of 20 μl of a 0.5% DNFB in 4:1 acetone/olive oil mixture on days 0 and 1(sensitization phase). Five days after sensitization, the mice were challenged on day 6 with application of 20 μl of a 0.5% DNFB in 4 : 1 acetone/olive oil mixture to the left inner and outer surfaces of the mice (elicitation phase). Ear thickness measurements of both the treatment and control/blank groups were taken with an electronic digital caliper at 24, 48 and 72 h after challenge and the response quantitated as the difference in the thickness of the challenged ear.

### Statistical analysis

Data are expressed as means ± S.E.M. One-way ANOVA or Student's *t*-test was used to determine the significance of difference; a value of *p* < 0.05 was considered statistically significant.

## SUPPLEMENTARY MATERIALS FIGURES AND TABLES


