# Supplementary material for: Isoliquiritigenin suppresses human T Lymphocyte activation via covalently binding cysteine 46 of IκB kinase
Source: Oncotarget. 2016 Sep 10;8(21):34223–35. doi: 10.18632/oncotarget.11934 (PMC5470962; doi:10.18632/oncotarget.11934)
Supplement: Supplementary file 1 [file oncotarget-08-34223-s001.pdf]

Isoliquiritigenin suppresses human T Lymphocyte activation *via* covalently binding cysteine 46 of IκB kinase

Supplementary Materials

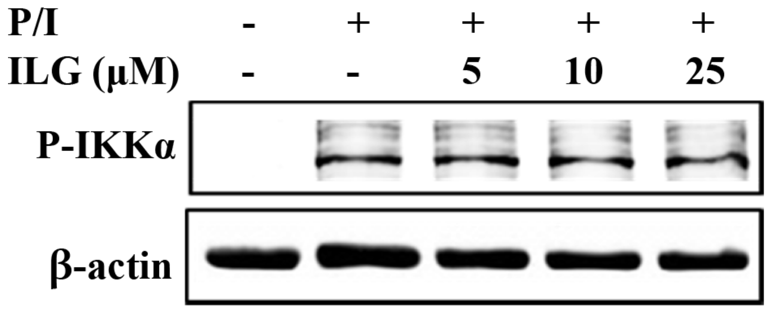

Supplementary Figure S1: The effect of ILG on IKKα phosphorylation in human T cells stimulated by P/I. Data are representative of three independent experiments.

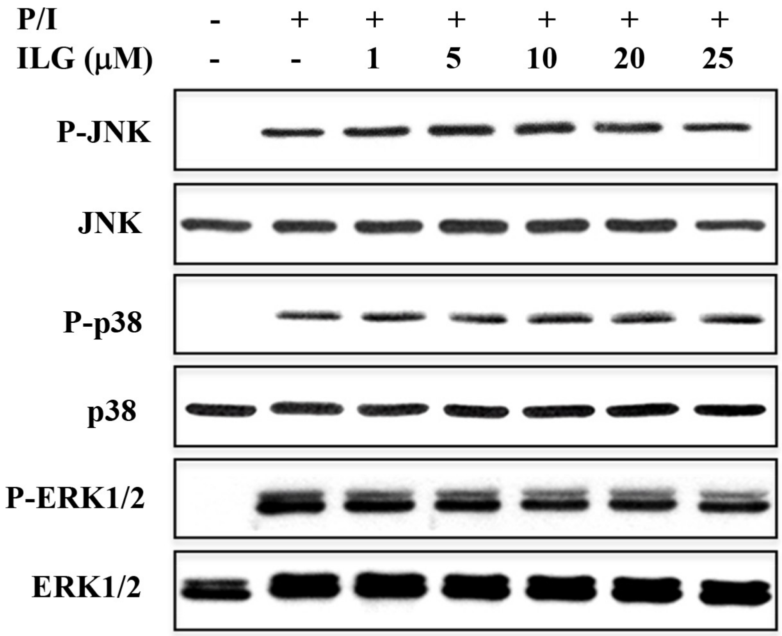

Supplementary Figure S2: The effect of ILG on MAPK phosphorylation in human T cells stimulated by P/I. Data are representative of two independent experiments.

Supplemenatry Table S1: The effect of ILG on the ear edema of the IKKβC46A transgenic mice and wild-type littermates with DTH

Group	*n*	24 h	%*I*	48 h	%*I*	72 h	%*I*
		ΔT ± S. E. M.		ΔT ± S. E. M.		ΔT ± S. E. M.	
Vehicle treatment_IKKβ ^{wt}	10	209.00 ± 10.16		256.00 ± 16.28		315.00 ± 12.22	
ILG 0.75 mg/ear_IKKβ ^{wt}	9	137.78 ± 6.19***	34.1	180.00 ± 15.09**	29.7	253.33 ± 19.65	19.6
DEX 0.025 mg/ear_IKKβ ^{wt}	7	145.71 ± 7.19**	30.3	148.57 ± 17.10**	42.0	157.14 ± 30.45***	50.1
Vehicle treatment_IKKβ^{C46A}	10	219.00 ± 13.37		273.00 ± 20.71		331.00 ± 10.48	
ILG 0.75 mg/ear_IKKβ^{C46A}	10	206.00 ± 11.08	5.9	266.00 ± 14.92	2.6	309.00 ± 15.60	6.6
DEX 0.025 mg/ear_IKKβ^{C46A}	10	140.00 ± 8.69***	36.1	150.00 ± 13.82***	45.1	153.00 ± 20.82***	53.8

Edema is expressed as the increase in ear thickness (ΔT in micrometer ± S.E.M.). Statistically significant differences compared to the vehicle treatment are expressed as *P <0.05 and **P <0.01; ***P <0.01, respectively. %I: Percentages of the inhibitory rate for each measurement were calculated from values for the treated group with respect to those for the vehicle treatment: ((Vehicle treatment-Compound treatment)/(Vehicle treatment)) ×100.
